# Alfabetización en salud bucal de los padres y su asociación con la salud bucal de sus hijos: una revisión

**DOI:** 10.21142/2523-2754-1203-2024-209

**Published:** 2024-09-17

**Authors:** Elizabeth Hidalgo-Mora, Katherine Jeanette Campos-Campos, Denisse Aguilar Gálvez, Roxana Patricia López-Ramos, Evelyn Alvarez Vidigal

**Affiliations:** 1 Especialidad en Odontopediatría, Departamento de Odontopediatría, Facultad de Ciencias de la Salud, Universidad Científica del Sur. Lima, Perú. milihm02@gmail.com Universidad Científica del Sur Especialidad en Odontopediatría, Departamento de Odontopediatría Facultad de Ciencias de la Salud Universidad Científica del Sur Lima Peru milihm02@gmail.com; 2 Departamento de Odontopediatría. Facultad de Ciencias de la Salud, Universidad Científica del Sur. Lima, Perú. campos.jeanette@gmail.com Universidad Científica del Sur Departamento de Odontopediatría Facultad de Ciencias de la Salud Universidad Científica del Sur Lima Peru campos.jeanette@gmail.com; 3 Posgrado en Odontopediatría, Facultad de Ciencias de la Salud, Universidad Científica del Sur. Lima, Perú. denisseaguilarg@hotmail.com , evelyn_vidigal@hotmail.com Universidad Científica del Sur Posgrado en Odontopediatría Facultad de Ciencias de la Salud Universidad Científica del Sur Lima Peru denisseaguilarg@hotmail.com evelyn_vidigal@hotmail.com; 4 Posgrado de Estomatología, Universidad Científica del Sur. Lima, Perú. roxana.lopezramos3@gmail.com Universidad Científica del Sur Posgrado de Estomatología Universidad Científica del Sur Lima Peru roxana.lopezramos3@gmail.com

**Keywords:** alfabetización en salud, salud bucal, niño, padres, health literacy, oral health, child, parents

## Abstract

**Introducción:**

La alfabetización en salud bucal (ASB) alude a las habilidades cognitivas y sociales que permiten a las personas obtener, procesar y comprender informaciones sobre el cuidado de la salud bucal que les permitan tomar decisiones correctas al respecto.

**Objetivo:**

Recolectar información actualizada referida a la asociación entre la ASB de los padres y la salud bucal de sus hijos.

**Materiales y métodos:**

Se realizó un proceso de búsqueda de publicaciones científicas en las bases de datos PubMed, Scopus, LILACS, SciELO y Google Scholar. Se utilizaron los descriptores “Oral Health Literacy”, “Health Literacy”, “Literacy in Dentistry”, “Oral Health”, “Oral Condition”, “Dental Caries”, “Early Childhood Caries”, “Children”, “Child, Preschool”, “Infant”, “Parents” y “Parenting”. Fueron incluidos artículos científicos publicados en idioma inglés, portugués y español, entre los años 2013 y 2023. Los estudios con diseños de estudios clínicos, caso-control, cohorte y transversales fueron incluidos. Las revisiones de literatura, sistemáticas y metaanálisis fueron excluidas.

**Resultados:**

Se identificaron 147 artículos. Luego, a través de la lectura de títulos, resúmenes y textos íntegros, dos investigadores entrenados seleccionaron las publicaciones, incluyéndose finalmente 14 artículos. Los artículos de esta revisión definieron a la ASB como predictor importante del cuidado de la salud bucal, siendo el REALD-30 el instrumento más utilizado. Además, la mayoría de los artículos reportaron la asociación entre el bajo nivel de ASB de los padres y el mal estado de salud bucal de sus hijos.

**Conclusión:**

Existe asociación entre el bajo nivel de ASB de los padres y el mal estado de salud oral de sus hijos. Además, el nivel socioeconómico de la familia se relaciona con los cuidados de la salud bucal de los niños, lo que conduce a que los padres, por desconocimiento y falta de recursos, adquieran actitudes desfavorables para sus hijos.

## Abstract (en)

**Introduction:**

Oral health literacy (OHL) refers to the cognitive and social skills that allow individuals to obtain, process and understand information about oral health care that allows them to make correct decisions.

**Objective:**

To collect updated information on the association between parents' oral health literacy and their children's oral health.

**Materials and Methods:**

A bibliographic search was carried out in the Scopus, PubMed, SciELO, LILACS and Google Scholar databases. The descriptors used were “Oral Health Literacy”, “Health Literacy”, “Literacy in Dentistry”, “Oral Health”, “Oral Condition”, “Dental Caries”, “Early Childhood Caries”, “Children”, “Child, Preschool”, “Infant”, “Parents” and “Parenting”. Scientific articles published in English, Portuguese and Spanish between 2013 and 2023 were included. Likewise, cross-sectional studies, cohort studies, case-control studies, and randomized clinical trials were included. Literature reviews, systematic reviews and meta-analyses were excluded.

**Results:**

147 articles were identified. Then, two trained researchers selected the publications through reading titles, abstracts and full texts. Finally, 14 articles were included. The articles included in this review defined OHL as an important predictor of oral health care, and the REALD-30 was the most used instrument. Furthermore, most of the articles reported the association between low level parents' OHL and their children's poor oral health status. **C**

**onclusion:**

There is an association between the low level of parents’ OHL and the poor oral health status of their children. Furthermore, the socioeconomic level of the family is related to children's oral health care, which leads parents, due to lack of knowledge and lack of resources, to adopt harmful attitudes towards their children.

## INTRODUCCIÓN

La alfabetización en salud bucal (ASB) es un término relacionado con las capacidades cognitivas y sociales que permiten a las personas recibir, procesar y entender las informaciones y servicios dirigidos al cuidado de la salud bucal, importantes para la toma asertiva de decisiones al respecto ^1^^-^^3^. Este concepto también se relaciona con determinantes sociales como las desigualdades en estratos socioeconómicos, que pueden limitar el acceso a servicios odontológicos ^4^^,^^5^. Además, este término enfatiza que el entendimiento funcional, aplicado y contextual de las conductas y cuidados de salud bucal son imprescindibles para lograr óptimas condiciones bucales ^6^^,^^7^.

Últimamente, la ASB ha ganado interés dentro de la odontología, pues está vinculada al grado de comprensión de las informaciones ofrecidas o al material educativo para programas de salud bucal. Una buena ASB implica brindar informaciones que se adapten al nivel de comprensión de las personas ^8^^,^^9^. Por este motivo, es esencial conocer el nivel de ASB para proporcionar información adecuada, utilizando los medios de acuerdo con las necesidades de los individuos, ya sea de los padres o sus hijos. 

Para medir el nivel de ASB se han desarrollado diferentes instrumentos, la mayoría de los cuales son cuestionarios, los cuales han sido utilizados en diversas investigaciones con el objetivo de determinar cuál es la relación entre el nivel de ASB y la salud bucal ^5^. Algunos estudios han reportado que los padres desempeñan un rol esencial en la prevención de la caries dental de sus hijos, por lo que existe una asociación entre los hábitos de cuidado y la salud bucal de ambos ^6^. 

Algunas investigaciones han demostrado que el bajo nivel ASB de los padres se asocia con una mayor experiencia de caries en sus hijos ^2^^,^^3^^,^^9^^,^^10^. Sin embargo, una revisión sistemática ha reportado que los resultados son aún contradictorios, pues, en relación con la asociación entre la ASB de los padres y las condiciones bucales de sus hijos, la evidencia es débil y los resultados no son concluyentes ^10^. 

Por otra parte, se ha reportado que los padres con ASB baja tendrían mayores probabilidades de tener hijos con alguna consecuencia clínica por lesiones de caries no tratadas ^11^. Asimismo, se ha encontrado asociación entre bajos niveles de ASB y ansiedad dental ^12^, un impacto negativo en la calidad de vida relacionada con la salud bucal (CVRSB) ^13^, y que las conductas de los padres a la hora de comer se asocian con la experiencia de caries de sus hijos ^14^. 

Con todo lo mencionado, es importante destacar que los profesionales de la salud desempeñan un rol importante en la mejora de la ASB, pues son ellos quienes brindan información sobre cuidados de salud bucal a los pacientes ^2^. Por este motivo, resulta primordial que ellos reciban información actualizada sobre alfabetización en salud bucal y se conviertan en agentes de cambio en la promoción de la salud bucal del paciente pediátrico ^2^^,^^9^. Por ello, la finalidad de esta revisión es recolectar informaciones actualizadas sobre la asociación entre la ASB de los padres y la salud bucal de sus hijos.

## METODOLOGÍA

**Figura 1:**
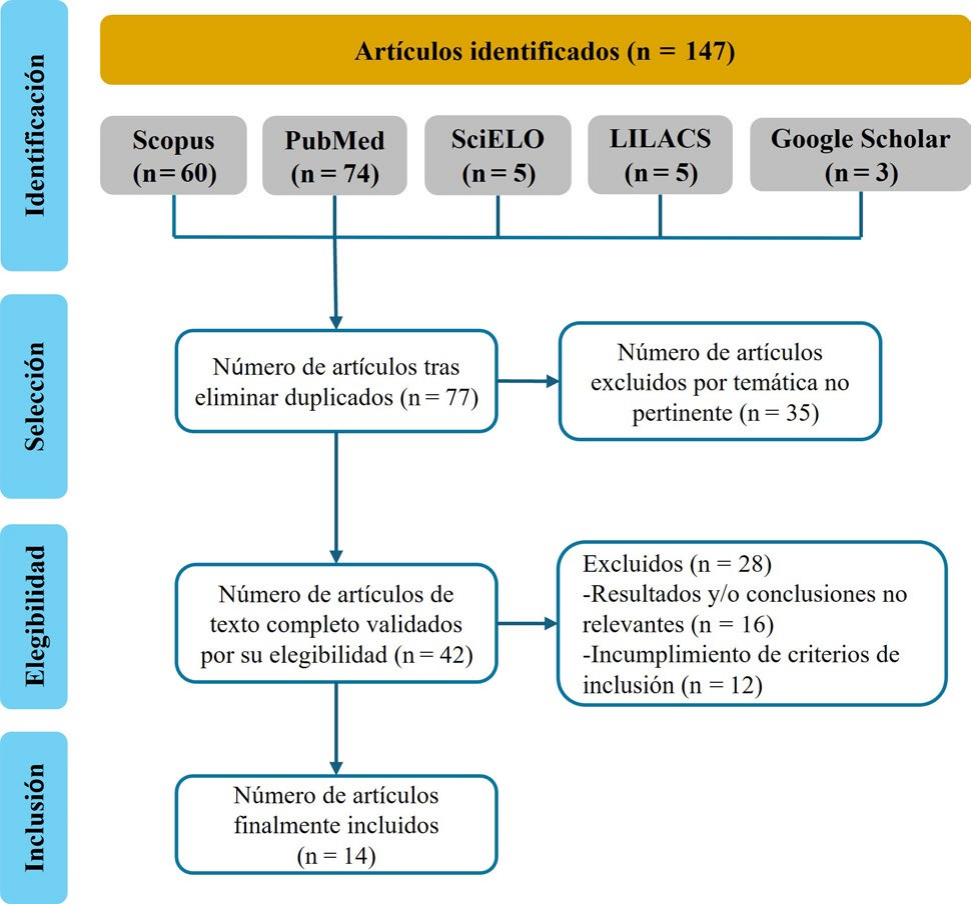
Flujograma de la estrategia de búsqueda y análisis de las publicaciones científicas.

La búsqueda de artículos fue realizada en las bases de datos PubMed, Scopus, LILACS, SciELO y Google Scholar. Se utilizaron los descriptores en inglés “Oral Health Literacy”, “Health Literacy”, “Literacy in Dentistry”, “Oral Health”, “Oral Condition”, “Dental Caries”, “Early Childhood Caries”, “Children”, “Child, Preschool”, “Infant”, “Parents” y “Parenting”. Para definir la estrategia de búsqueda se utilizaron los conectores booleanos AND y OR. Fueron incluidas publicaciones científicas publicadas entre los años 2013 y 2023, en idioma inglés, portugués y español, que evaluaron la asociación de la ASB de los padres con la salud bucal de sus hijos con edades de 0 a 9 años. Los estudios con diseños de estudios clínicos, caso-control, cohorte y transversales fueron incluidos; mientras que las revisiones de literatura, sistemáticas y metaanálisis fueron excluidas. Dos investigadores entrenados evaluaron independientemente los títulos y resúmenes de los 147 artículos identificados en un primer momento. Luego de eliminar los duplicados y leer los textos completos aplicando los criterios de elegibilidad, fueron incluidos 14 artículos. Todo este proceso de búsqueda se encuentra esquematizado en la Figura 1. 

## RESULTADOS

### Alfabetización en salud bucal

La alfabetización en salud (AS) es considerada un fuerte predictor de salud junto con la edad, situación laboral, ingresos económicos, nivel de instrucción y grupo étnico ^15^. Así, su ausencia es considerada un factor crítico, pues se trata de una importante estrategia para mantener a los individuos en condiciones saludables ^16^. Esto, en cierta forma, se refleja en reportes de personas con bajo nivel de AS, lo que impacta negativamente en su calidad de vida, pues son frecuentemente hospitalizadas, utilizan más servicios de urgencia y presentan más enfermedades crónicas, como la caries dental ^16^^-^^19^.

La literatura científica define a la alfabetización en salud bucal (ASB) como la facultad de recibir, procesar y entender informaciones relacionadas con la salud oral, las cuales son importantes para la correcta toma de decisiones ^(1-3, 20)^. Además, este término de ASB fue definido en 2010 como un instrumento importante para incentivar los cuidados de la salud bucal ^21^. En la presente revisión, todos los artículos incluidos coincidieron con estas definiciones enfatizando la asociación de los bajos niveles de ASB con el conocimiento deficiente de los padres sobre los cuidados de salud bucal, e incluso con los gastos elevados de atención por emergencias ^24^; sin embargo, aún se reportan resultados conflictivos, pues también se han encontrado reportes contrarios ^20^.

### Instrumentos para la medición de la alfabetización en salud bucal

Con la finalidad de investigar el nivel de ASB, se han desarrollado diversos instrumentos, el primero de los cuales es el Rapid Estimate Adult Literacy in Dentistry (REALD-30) ^22^. Existen alrededor de 13 instrumentos que pueden agruparse en tres categorías de acuerdo con la dimensión que evalúan, como identificación de las palabras, comprensión lectora y estimaciones numéricas, y conocimiento de conceptos ^23^. 

En la categoría de reconocimiento de palabras, podemos mencionar instrumentos como Rapid Estimate of Adult Literacy in Dentistry (REALD-99), Rapid Estimate of Adult Literacy in Medicine and Dentistry (REALM-D) y Rapid Estimate of Adult Literacy in Medicine and Dentistry (REALMD-20). En relación con la categoría de comprensión de lectura y cálculo numérico, tenemos el Test of Functional Health Literacy in Dentistry (TOFHLiD), Oral Health Literacy Instrument OHLI, Oral Health Literacy Questionnaire (OHLQ), Oral Health Literacy Assessment (OHLA-E) y Hong Kong Oral Health Literacy Assessment Task for Paediatric Dentistry (HKOHLAT-P). Finalmente, en la categoría de conocimiento de conceptos tenemos al Comprehensive Measure of Oral Health Knowledge CMOHK y Health Literacy in Dentistry (HeLD). Cabe mencionar que muchos de estos instrumentos han sido traducidos a otros idiomas, siendo el REALD (versiones 30 y 99) el más adaptado ^23^. 

En la presente revisión de literatura, la mitad de los artículos incluidos utilizaron el instrumento REALD-30 en sus versiones adaptadas transculturalmente correspondientes a cada país ^4^^,^^9^^,^^12^^,^^14^^,^^24^^,^^28^^,^^29^, lo que coincide con lo reportado respecto de su amplio uso en investigaciones ^23^. Otros estudios incluidos utilizaron instrumentos como OHLA ^3^^,^^6^, OHLI ^2^ y BOHLAT-P ^11^^,^^25^, correspondientes a la categoría de comprensión de lectura y cálculo numérico, así como el instrumento HeLD-ID, perteneciente a la categoría de conocimiento de conceptos ^26^^,^^27^.

### Alfabetización en salud bucal de los padres y su asociación con la salud bucal de los niños

Esta revisión de literatura incluyó 14 artículos publicados en los últimos diez años, de los cuales uno fue un estudio de cohorte prospectivo, dos fueron estudios poblacionales-transversales y 11 fueron transversales. Estos estudios evaluaron la asociación de la ASB de los padres con la salud bucal de sus hijos, además de otras variables (Tabla 1).

**Tabla 1:** Estudios que abordan la asociación entre la ASB de padres y la salud bucal de sus hijos incluidos en la revisión de literatura

AUTOR, AÑO Y PAÍS	MUESTRA	EDAD ^(^padres y/o niños)	TIPO DE ESTUDIO	INSTRUMENTO	CONCLUSIONES
Vann *et al*. 2013 ^24^ EE. UU.	n = 1132 (niños/padres)	≤60 meses (niños) 18-65 años (padres)	Cohorte-prospectivo	REALD-30	Los gastos de la atención odontológica de emergencia fueron muy elevados en los niños con padres con bajo nivel de ASB.
Bridges *et al*. 2014 ^4^ China	n = 301 (niños/padres)	5 años	Transversal	HK-REALD-30 / Hong Kong Oral Health Literacy Assessment Task for Pediatric Dentistry HK-OHLAT-P	La ASB de los padres se asoció con la salud bucal de sus hijos.
Shin *et al*. 2014 ^12^ EE. UU.	n = 187 (padres)	37 años	Transversal	REALD-30	Los factores que conducen a una mala salud bucal de los niños están interrelacionados e incluyen un bajo nivel de ASB y ansiedad dental de los padres.
Khodadadi *et al*. 2016 ^6^ Irán	n = 384 (niños/padres)	21-84 meses	Transversal	Oral Health Literacy Adults Questionnaire (OHL-AQ)	Padres con bajo nivel de ASB tienen hijos con mayor número de lesiones de caries y menor cantidad de dientes tratados.
Montes *et al*. 2019 ^9^ Brasil	n = 415 (padres)	4-5 años	Estudio poblacional-Transversal	BREALD-30	Existe una mayor prevalencia de lesiones de caries dental no tratada en niños preescolares cuyos padres tenían un bajo nivel de ASB.
Adil *et al*. 2020 ^2^ Malasia	n = 230 (niños/padres)	3-6 años	Transversal	Oral Health Literacy Instrument (OHLI-M)	La baja ASB los padres se asoció significativamente con la experiencia de caries de sus hijos preescolares.
Martins *et al*. 2021 ^11^ Brasil	n = 449 (niños/padres)	4-6 años	Transversal	BOHLAT-P	Los padres con ASB más baja tenían mayores probabilidades de tener hijos con al menos una consecuencia clínica de caries dental no tratada.
Emadzadeh *et al*. 2021 ^3^ Irán	n = 205 (niños/padres)	3-6 años	Transversal	Oral Health Literacy for Adults Questionnaire (OHL-AQ)	La baja ASB de los padres y vivir con varios miembros familiares se asoció con alta prevalencia de caries dental en sus hijos.
Sowmya *et al*. 2021 ^28^ India	n = 449 (niños/padres)	2-6 años	Transversal	REALD-30	El comportamiento de la madre y la baja ASB se asociaron significativamente con la experiencia de caries en sus hijos.
Wang *et al.* 2022 ^29^ China	n = 406 (niños/padres)	4-7 años	Transversal	HKREALD-30 / HKREALD-30-Understand	La ASB de los padres afecta su propio cuidado de la salud bucal y la salud bucal de sus hijos.
Rachmawati *et al*. 2022 ^27^ Indonesia	n = 306 (niños/padres)	5-6 años	Transversal	HeLD-ID	Existe una asociación entre la ASB de los padres y la experiencia de caries de sus hijos. Sin embargo, no se encontró correlación con la CVRSB de los niños.
Moriyama *et al*. 2022 ^14^ Brasil	n = 630 (niños/padres)	2-4 años	Transversal	BREALD-30	La ASB de los padres no se asoció con la experiencia de caries dental en sus hijos. Sin embargo, los comportamientos de los padres a la hora de comer se asociaron con la experiencia de caries dental en sus hijos.
Ferreira *et al*. 2023 ^25^ Brasil	n = 449 (niños - padres)	4-6 años	Estudio poblacional-transversal	BOHLAT-P	Niños preescolares con lesiones de caries cavitadas y con padres con una alta ASB utilizaron con mayor frecuencia los servicios de atención de salud bucal.
Gomathi *et al*. 2023 ^26^ India	n = 350 (niños -padres)	3 a 9 años	Transversal	HeLD	El nivel de ASB de los padres se asoció significativamente con el estado de salud bucal de sus hijos.

El nivel de ASB de los padres se asoció significativamente con el estado de salud bucal de sus hijos.

Diversos estudios han reportado que los bajos niveles de ASB se asocian con el escaso conocimiento e inadecuados cuidados de la salud bucal ^20^, que podrían involucrar numerosos gastos por la atención odontológica. Al respecto, Vann *et al*. ^24^ realizaron un estudio de cohorte prospectivo en 1132 niños y padres con la finalidad de evaluar si la ASB de estos últimos influye en los gastos relacionados con la atención odontológica de sus hijos. Dicho estudió utilizó el instrumento REALD-30 y encontró que los gastos en atenciones de emergencia fueron sumamente elevados en aquellos niños cuyos padres tenían un bajo ASB. En contraparte, un estudio de base poblacional realizado por Ferreira et al. ^25^ evaluó el impacto de la caries de la primera infancia y la ASB de los padres. Los autores aplicaron el cuestionario BOHLAT-P y hallaron que los niños preescolares con lesiones de caries cavitadas y de padres con una mayor capacidad de comprensión sobre los cuidados de salud bucal utilizaron servicios odontológicos con mayor frecuencia.

Con respecto a la asociación de la ASB de los padres y las condiciones bucales de sus hijos, una investigación con diseño transversal, realizada por Bridges *et al*. ^4^, evaluó esta asociación en 301 de niños de 5 años y sus padres utilizando los instrumentos HKREALD-30 y HKOHLAT-P. Los resultados mostraron que los niños con alta prevalencia de caries y presencia de placa visible tenían padres con menor nivel de ASB, y que el HKOHLAT-P fue el más sólido para determinar esta asociación. Por otro lado, Gomathi *et al*. ^26^ también evaluaron esta asociación en 350 niños de 3 a 9 años y sus respectivos padres. A diferencia del anterior estudio, se encontró que las madres con alto nivel de ASB tenían sus hijos con mejores condiciones bucales; además, los autores utilizaron el instrumento Health Literacy in Dentistry scale (HeLD). En ambos estudios, algunas variables socioeconómicas, como ingreso mensual, grado de instrucción de los padres y situación laboral, también se asociaron con las condiciones bucales de los niños; así, familias con mejores condiciones socioeconómicas tenían hijos con mejores condiciones bucales ^4^.

Con relación a la caries dental, diversos estudios transversales han reportado asociación entre la baja ASB de los padres y la mayor experiencia de lesiones de caries en sus hijos. Al respecto, Khodadadi *et al*. ^6^ evaluaron a 384 niños 21 a 84 meses de edad y sus padres, aplicando el cuestionario Oral Health Literacy Adults Questionnaire (OHL-AQ), y encontraron una relación significativa entre vivir en un área rural, tener baja ASB y presentar menos lesiones de caries tratadas. De igual forma, dos estudios transversales que evaluaron a niños de 3 a 6 años encontraron que la baja ASB de los padres se asoció significativamente con la mayor prevalencia de caries dental ^2^^,^^3^. Además, en uno de estos estudios, la convivencia con varios miembros familiares también estuvo asociada con la mayor prevalencia de caries ^3^. Cabe resaltar que en ambos estudios se utilizaron instrumentos diferentes, como el Oral Health Literacy Instrument (OHLI-M) ^2^ y el Oral Health Literacy for Adults Questionnaire (OHL-AQ) ^3^.

De la misma forma, una investigación poblacional desarrollada por Montes *et al*. ^9^, en 415 niños de 4 a 5 años y sus padres, examinó la asociación entre la ASB de los padres y dientes con lesiones de caries no tratadas en sus hijos. En este trabajo, los autores utilizaron el instrumento BREALD-30 y hallaron una mayor prevalencia de lesiones de caries dental no tratada en niños cuyos padres tenían un bajo ASB. En esa línea de objetivos, Martins *et al*. ^11^ desarrollaron un estudio transversal en 449 niños de 4 a 6 años y sus padres, con el propósito de examinar la asociación entre la ASB de los padres y las lesiones de caries no tratada y sus consecuencias clínicas en sus hijos. El cuestionario BOHLAT-P fue aplicado y los autores concluyeron que los padres con ASB más baja tenían mayores probabilidades de tener hijos con al menos una consecuencia clínica de caries dental no tratada. 

Por otro lado, existen estudios que han evaluado la asociación de otras variables. Así, Rachmawati *et al*. ^27^ desarrollaron un estudio transversal en 306 niños de 5 a 6 años y sus padres con la finalidad de evaluar la correlación entre la ASB de los padres, la prevalencia de caries dental y la CVRSB de sus hijos. Los investigadores aplicaron el instrumento HeLD-ID y hallaron una asociación entre la ASB de los padres y el número de lesiones de caries de sus hijos; sin embargo, no encontraron correlación con la CVRSB de los niños. 

Shin *et al*. ^12^ exploraron la asociación entre la ansiedad dental de los padres y la alfabetización en salud bucal, y la asociación entre estas variables con las condiciones bucales de sus hijos. Los autores utilizaron el cuestionario REALD-30 y encontraron una correlación la ASB de los padres y la ansiedad dental de estos, donde una buena forma de comunicarse con los padres con bajo nivel de ASB puede reducir su nivel de ansiedad. Además, los resultados señalaron que, en los niños socioeconómicamente desfavorecidos, la baja ASB de los padres y la ansiedad dental son factores que contribuyen a malas condiciones de salud bucal. 

Asimismo, un estudio transversal realizado en 449 madres y sus respectivos hijos en edades de 2 a 6 años tuvo como objetivo evaluar la asociación entre el comportamiento de las madres, en términos de conocimiento, las actitudes y prácticas relacionadas con el cuidado bucodental, la ASB de la madre y el número de lesiones de caries dental de sus hijos. Al igual que el estudio de Shin *et al*. ^12^, el REALD-30 fue el instrumento utilizado. Los resultados de esta investigación mostraron que las madres con mejores prácticas, actitudes y conocimientos sobre los cuidados de la salud bucal tenían hijos con mejores condiciones bucales; por tanto, el comportamiento y la baja ASB de la madre se asociaron significativamente con la experiencia de caries en niños.

Considerando que la caries dental está muy relacionada con el consumo de azúcares, Moriyama *et al*. ^14^ ejecutaron una investigación transversal con el propósito de verificar cómo influye el comportamiento de los padres en el desarrollo de lesiones de caries en sus hijos. Los investigadores evaluaron el ASB y comportamiento de los padres durante las comidas de sus hijos. En esta investigación se aplicó el cuestionario BREALD-30 y los autores hallaron que, aunque la ASB de los padres no se asoció con la caries dental en los niños, los comportamientos de los padres a la hora de comer sí mostraron asociación con la caries dental.

Finalmente, desde otra perspectiva, Wang *et al*. ^29^ desarrollaron un estudio transversal en 406 padres con hijos de 4 a 7 años para explorar el rol y la asociación de la ASB en la salud bucal de los padres y sus hijos. Los cuestionarios HKREALD-30 y HKREALD-30-Understand fueron utilizados y, como resultado, los autores reportaron que una baja ASB de los padres afecta su propio cuidado de la salud bucal y el de sus hijos. 

## DISCUSIÓN

El objetivo de esta investigación fue recopilar información actualizada sobre la asociación entre la ASB de los padres y la salud bucal de sus niños. Es importante considerar que, de los 14 artículos incluidos en esta revisión de literatura, la mayoría demostró dicha asociación. No obstante, en un único artículo no se encontró asociación entre estas variables, y se observó un sesgo socioeconómico, al tratarse de un estudio realizado en estratos sociales más elevados, por lo que este se considera un indicador más favorable de alfabetización en salud bucal ^14^. 

Al respecto, uno de los factores más destacados asociados con la caries dental durante la infancia es el factor sociodemográfico ^16^. El grado de ASB está influenciado por muchos factores sociales, siendo determinantes importantes para la salud de las personas, así como el de sus hijos. Estos factores abarcan tipo de vivienda, número de hijos, desempleo, entre otros ^30^. Dicha asociación se evaluó en varios de los artículos seleccionados y se halló una asociación importante entre el bajo nivel de ASB y el hecho de vivir en áreas rurales ^27^. Estos datos son confirmados con estudios donde se observa que nivel de ASB de los padres tiende a ser más bajo cuando tienen un estatus socioeconómico más pobre o un bajo grado de instrucción ^12^^,^^26^^,^^30^.

Por otro lado, cuando se asoció con el número de miembros de la familia, se observó que los padres con muchos hijos presentan un menor nivel de ASB, y sus hijos tienen un mayor número de lesiones de caries ^15^^,^^16^. Esta relación coincide con lo encontrado por Velasco *et al*. ^31^, quienes afirmaron que existe una correlación negativa entre la salud bucal de los niños, la baja ASB de los padres y un mayor número de hermanos, posiblemente debido a que tienen menos tiempo para dedicarse a cada uno de sus hijos.

El desempleo fue otro factor analizado y los resultados revelaron una correlación entre este, el bajo nivel de alfabetización y el alto número de piezas dentarias afectadas por caries ^32^. Estos hallazgos son consistentes con los encontrados en una revisión sistemática previa, donde se reportó que factores como el bajo nivel socioeconómico de la familia y los hábitos de salud oral deficientes de los padres contribuyen al desarrollo de la caries dental ^33^ en niños de 5 años. Otros investigadores ^34^ también encontraron que los niveles de ingresos están asociados con la ASB, lo que posiblemente se deba a la estrecha relación entre el acceso a la educación y los ingresos familiares. Se ha demostrado que tanto los ingresos familiares como la educación tienen un impacto en la gravedad de la caries dental ^20^, eso debido a que muchos padres tal vez no entiendan completamente las instrucciones que les proporcionan los profesionales de salud ^7^^,^^10^ y que la ASB aumenta con los niveles de instrucción, ya que los padres con estudios de primaria y los desempleados tienden a tener más bajos niveles de esta.

Cabe resaltar que este dato no coincide con lo encontrado por Macedo *et al*. ^34^, quienes informaron que los participantes con un bajo nivel de ASB tenían un ingreso medio, eran propietarios de vivienda propia y tenían secundaria completa. Por otro lado, el costo promedio que destinan los padres de niños sanos a las atenciones preventivas es considerablemente menor en comparación con el costo promedio cuando el niño está enfermo, y la mayor prevalencia de niños que nunca han ido al dentista está muy ligada al menor nivel de ASB de sus padres ^35^. Estos datos confirman los reportes que señalan que los padres con baja ASB tienen más probabilidades de tener hijos con un estado de salud bucal deficiente ^2^^,^^7^^,^^9^^,^^10^. 

En esta revisión de literatura, se encontró que los padres con niveles bajos de ASB enfrentan gastos totales significativamente más elevados, debido a la necesidad de buscar atención odontológica de emergencia en repetidas oportunidades para sus hijos ^24^. Además, encontró que los padres con un bajo estatus socioeconómico y bajo ASB tienen hijos con un mayor número de caries no tratadas ^6^^,^^9^^,^^10^^,^^14^, lesiones pulpares y abscesos ^9^, pero que esto se relacionado más con la baja ASB que con la falta de recursos económicos ^3^^,^^6^^,^^9^^,^^36^. Esto puede contrastarse con investigaciones previas, las cuales indican que las personas con un bajo ASB utilizan menos los servicios odontológicos, no por falta de necesidad, sino porque no comprenden la importancia de llevar a sus hijos a recibir atención dental temprana o porque desconocen las consecuencias a largo plazo que esto puede acarrear ^14^^,^^37^^-^^39^.

Se reconoce que las madres desempeñan un papel clave en todas las intervenciones tempranas y eficaces relativas a sus hijos, pues los hábitos de higiene bucal se establecen durante los primeros años de vida, y ellas son los modelos de aprendizaje, debido a la disposición para vincularse con actividades preventivas que ayuden a mejorar la salud de sus hijos ^13^^,^^27^. Por ello, los niveles de ASB de las madres están relacionados con la salud bucal de sus hijos ^3^^,^^40^. De esta forma, mejorar la ASB de las madres podría fortalecer su capacidad para promover la salud bucal, y de esta manera mejorar la salud dental de sus hijos y reducir las disparidades ^41^. Este argumento se debe a que las madres de niños de bajos recursos, por lo general, tienen bajo grado de instrucción, lo que genera condiciones de vida precarias y habilidades de comunicación deficientes. Esto, a su vez, afecta negativamente tanto la salud bucal de las madres como las de sus hijos ^28^. Una madre con ASB alto utiliza métodos preventivos en salud bucal para sus hijos con mayor frecuencia ^27^^,^^42^. Dado que las madres juegan un papel crucial en todas las intervenciones tempranas con sus hijos, es fundamental tenerlas en cuenta al diseñar e implementar políticas destinadas a mejorar la salud bucal de esta diada ^17^, adecuados a su grado de instrucción ^41^, sobre todo en aquellas madres trabajadoras que tienen menos tiempo de calidad con sus hijos ^35^.

Se ha demostrado que la ansiedad dental impacta negativamente en la salud bucal y que los pacientes con este estado emocional generalmente requieren más tiempo de tratamiento odontológico ^43^. Un hallazgo importante es la relación entre nivel de ASB, el estatus socioeconómico y la ansiedad de los padres, siendo menos probable que los padres con baja ASB lleven a sus hijos al dentista ^44^; por ello, es importante responder a sus inquietudes de manera que disminuya su nivel de ansiedad ^12^. Este resultado se refuerza con dos estudios previos que encontraron que los altos niveles de ansiedad dental de los padres estaban relacionados con el bajo grado de ASB, pero que estos se hallaban condicionados por la edad y el género del niño ^45^^,^^46^. Por otra parte, las madres con un alto nivel de ASB tienen hijos que sufren menos de enfermedad de caries, mayor conocimiento sobre salud infantil y buenas prácticas preventivas, que garantizan un buen cuidado de la salud bucal ^46^.

Además, se ha reportado que los padres que poseen información sobre su propia salud bucal son aquellos que tienen hijos mejor alimentados y con mejor salud bucal ^47^. La revisión sistemática ^33^ que analiza la influencia del entorno familiar en la salud oral de los niños destaca la importancia de la familia, los cuidadores y los profesionales de la salud en el establecimiento de un nivel adecuado de ASB y comportamientos saludables. Esto nos demuestra que los cambios positivos en el estado de salud y los comportamientos de salud de los niños se verá desarrollado a través de la formación de ASB de los padres ^45^. 

Por todo lo expuesto, podemos decir que conocer el nivel de ASB de los padres es muy importante en el momento de planificar estrategias e implementar programas para la promoción en salud dirigidos a los padres, y conseguir así motivarlos a preservar la salud bucal de sus hijos.

### Limitaciones

Si bien la presente revisión incluyó publicaciones científicas relevantes y actualizadas sobre la asociación de la ASB de los padres y la salud bucal de sus hijos, la extracción de informaciones y análisis cualitativo no presenta una rigurosidad que evalúe la validez de los resultados encontrados, por lo que la extrapolación de los resultados es limitada. 

## CONCLUSIONES

Existe una asociación entre el bajo nivel de ASB de los padres o cuidadores y el mal estado de salud bucal de sus niños. Asimismo, el nivel socioeconómico de la familia se relaciona con los cuidados de la salud bucal de los hijos, lo que conduce a que los padres, por desconocimiento y falta de recursos, adquieran actitudes desfavorables para sus hijos.

## Fuente de financiamiento:

Autofinanciado
